# Chronic in situ tissue cooling does not reduce lignification at the Swiss treeline but enhances the risk of ‘blue’ frost rings

**DOI:** 10.1007/s00035-023-00293-6

**Published:** 2023-02-28

**Authors:** Christian Körner, Armando Lenz, Günter Hoch

**Affiliations:** Department of Environmental Sciences, Botany, University of Basel, Schönbeinstrasse 6, 4056 Basel, Switzerland

**Keywords:** Alpine treeline, Cell wall, Conifers, Freezing tolerance, Histology, Lignin, Low temperature, Wood anatomy, Xylogenesis

## Abstract

In their 2013 paper, Lenz et al. illustrated how trees growing at the low-temperature limit respond to a chronic in situ warming or cooling by 3 K, by employing Peltier-thermostated branch collars that tracked ambient temperatures. The micro-coring-based analysis of seasonal tree ring formation included double-staining microtome cross sections for lignification, but these data had not been included in the publication. In this short communication, we complement these data, collected in 2009 at the Swiss treeline, and we show that a 3 K cooling that corresponds to a 500–600 m higher elevation, had no influence on lignification. However, when a frost event occurred during the early part of ring formation, the 3 K cooling produced a blue (non-lignified) layer of cells, followed by normally lignified cells for the rest of the season. Hence, the event did not affect the cambium, but interrupted cell wall maturation in cells that were in a critical developmental stage. We conclude, that chronic cooling does not affect lignification at treeline, but it increases the risk of frost damage in premature xylem tissue.

## Treeline formation and xylogenesis

Two entirely independent assessments of temperatures at undisturbed, climatic treelines (Körner and Paulsen 2004; Paulsen and Körner 2014) revealed that the global treeline position tracks a c. 6 °C seasonal mean temperature. This temperature threshold does account for a 0.3–0.5 K warming that had already occurred in extratropical treelines, when these assessments have been conducted (while the shift in treeline position did not yet match this warming trend). Once this isotherm had been identified—with disturbances and local peculiarities such as lack of soil or suitable species ruled out—the mechanistic explanation of the global treeline phenomenon shifted from carbon relations to growth control (Körner 1998, 2012, 2021). There is no indication that trees living at the cold edge of the fundamental niche of the life form ‘tree’ are carbon (photosynthesis)-limited. Because photosynthesis runs at high rates at temperatures so low that meristematic activity is halted, non-structural carbon reserves (carbohydrates, lipids) of trees tend to increase as one approaches the low-temperature tree limit (Hoch and Körner 2012). All this arouse interest in cellular processes during tree ring formation at the cold limit of tree growth (e.g., Vaganov et al. 2006; Gruber et al. 2009; Moser et al. 2010; Cuny et al. 2019; Cabon et al. 2020). In these works, the dynamics of tree ring formation were brought into a concurrent climate context. Rossi et al (2007) found a 5.6–8.5 °C air temperature range for a low-temperature threshold for xylogenesis. Such thresholds are pragmatic approximations that do not exactly identify the temperature that prevents tissue formation because the decline in such formative processes is asymptotically approaching zero. Hence, any threshold is a matter of time resolution and depends on the scale of growth rate assessment.

Most importantly, what actually happens at such critically low temperatures at cell wall level is still poorly understood despite the excellent understanding of the processes involved at mild and warm temperatures (Donaldson 2001; Rathgeber et al. 2016; Cantreau and Tuominen 2022), but there seems to be no difference in low-temperature effects between shoot and root or herbaceous and woody species (Alvarez-Uria and Körner 2007; Körner 2008; Schenker et al. 2014; Nagelmüller et al. 2017). Hence, the direct impact on cell formation appears to be universal for cold-adapted plants, with no plant growing below 0 °C and the rates extremely low below 5 °C, and linear responses between 10 and 18 °C for treeline trees (Cuny et al. 2019). The reason why trees are limited at lower elevation or latitude (thus, forming a treeline) than shrubs and herbs/grasses is related to their aerodynamic coupling to ambient air temperature, while smaller stature plants decouple, hence experience a much warmer microclimate than trees at treeline, when the sun is out (Körner 2012; see Fig. 4 in Körner 2021).

**Fig. 1:**
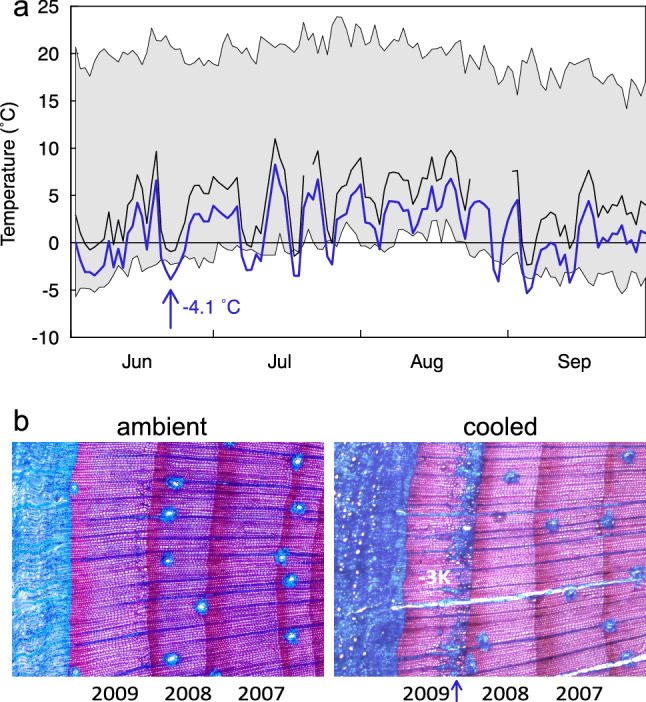
Cambial temperatures during the 2009 experiment at treeline on Stillberg, Davos. Black line for reference cambium, blue line for Peltier-cooled ( – 3 K) cambium (means for all 6 treated branches), with the blue arrow pointing at the fatal frost event on 22 June 6–7 h a.m. The envelope shows the range of extremes (minima and maxima) of air temperature for the period 1975–2007 at Stillberg (gray shade; Lenz et al. 2013). The microscope images show cross sections of control (ambient) and treatment ( 3 K cooled) branches, with the blue arrow pointing at the non-lignified zone (blue frost ring) related to the – 4.1 °C freezing during the most critical period of tracheid formation

To better understand xylogenesis at the low-temperature limit of tree growth, Lenz et al (2013) designed an experiment with 12 individuals of *Pinus uncinata* at the Swiss treeline at Stillberg near Davos, in which cambium temperature was manipulated. The experiment permitted to study tissue formation under a cold or warm collar (6 trees each), and compare it with untreated tissue responses at ambient temperatures. We employed computer-controlled Peltier collars that were set to track temperatures of untreated control branches by plus or minus 3 K, corresponding to a climatic shift in meristematic temperature by 500–600 m below (warming) or 500–600 m above (cooling) of the current treeline position. The treatment started at snowmelt and ended after xylogenesis was completed in autumn (see Fig. 1).

## Does in situ cooling affect lignification?

Lenz et al. (2013) showed that the number of new, undifferentiated cell rows during the most active, earliest part of cambial activity in June (type 4–5 cells by Rathgeber et al. 2016) correlated with the total (final) number of cell rows produced per tree ring. Warming or cooling affected the early wood/late wood ratio only. Chronic warming extended the early wood formation into the late season, similar to what was observed in low elevation pines by Piermattei et al. (2015) at the end of an exceptionally warm summer. Since the chronic warming/cooling treatment did not affect the degree of tree ring lignification, this was, unfortunately, not felt worth reporting in the original paper. Here, we complement this anatomical assessment by reporting the missing lignification data.

Although the biochemical process of xylem lignification had been found to continue at constant 0 °C (Körner et al. 2019), spot findings of insufficient tree ring lignification at or near treeline nourished the debate whether lignification could add to the overall growth constraints imposed by low temperature (for an early discussion see Körner 2008). It is known that monolignols are synthesized in midwinter (the ‘winter boost of monolignols’) with polymerization requiring e.g., peroxidases, which become available only once the cambium becomes active (Cantreau and Tuominen 2022). Since lignin stains red and cellulose stains blue with the most popular dyes, the term ‘blue ring’ came in use (for a literature review see Piermattei et al. 2015). These observations included (1) so called ‘frost rings’, zones of early wood that remain non-lignified (blue) and often show damaged or deformed cells after exceptional freezing events (Kaennel and Schweingruber 1995), (2) incomplete or missing lignification at the end of late wood differentiation due to bad autumn weather (Piermattei et al. 2015), and (3) generally weak lignification as might occur during exceptional summer weather that had been attributed to historical volcanic eruptions (Büntgen et al. 2020). Two recent publications speculated about a more general role of lignification for controlling plant limits, including tree limits, but these papers (including the idea that alpine herbs are small because they cannot produce lignin) provide no substance for such reasoning (Crivellaro and Büntgen 2020, Crivellaro et al. 2022). Here, we provide experimental evidence for the consequences of cooling the tree cambium at the cold edge of tree occurrence.

In brief, Safranine-Astrablue stained microtome cuttings of micro-cores collected in this experiment were mounted to microscope slides with a hydrophobic mounting medium (see Lenz et al. 2013). These slides could be retrieved now and re-inspected. In the original paper, the red Safranine staining was only employed to identify full lignification of tissues to ascertain maturity.

In no case did chronic, full season cooling or warming by 3 K at the low-temperature tree limit lead to a visible difference in lignification compared to controls. In other words, changing the temperature by plus or minus 3 K compared to ambient during xylogenesis, did not affect visual ‘redness’ in the xylem tissues produced at treeline during that experimental season. Hence, thermally shifting these trees upslope by 500–600 m produced no traces of a lignification problem.

However, as we now re-inspected these microscope slides, we became aware of a seemingly overlooked blue zone of disturbed cells in the earliest part of the 2009 tree ring in two of the cooled branches. In these samples, a number of cells in the first c. 10 rows of cells produced in the 2009 season did not lignify (Fig. 1). Re-inspecting the temperature records collected under each Peltier-collar revealed that the two branches experienced slightly colder temperatures ( – 4.1 °C) than the other 4 branches (mean across all – 3.9 °C) in the morning hours of 22 June 2009, when the close-by weather station reported – 3.6 °C. By chance, we appeared to have operated these thermostats just at the limit of freezing tolerance for such immature, thin-walled cells. The degree of subsequent weakening (or not continuing differentiation) in these cells caused holes in the microtome slices, with the seemingly undamaged branches showing only very light irregularities in the corresponding cell layer and red staining. After this ‘event’, all follow-up cell rows in the branches with a frost ring remained undistinguishable from controls, that is, they developed normal size and became fully lignified, despite the continuous experimental cooling. Later frost events (Fig. 1) had no effect. The somewhat fragmented blue zone is wider (covers a broader range of cell rows) near ray tissue. It almost looks like that the critical ice formation nucleated in ray tissue and spread from there. There are a number of inter-ray fields with cells that remained undamaged and also lignified.

To the extent the summer 2009 is representative (see below), we conclude that (1) chronic cooling by 3 K does not affect lignification at the alpine treeline; (2) a freezing event can interrupt cell wall differentiation in young tracheid cells (cells presumably become inactivated, if not killed); (3) the cambium itself remained undamaged by the frost event and produced vital cells thereafter, that became normally lignified. These freezing effects support the assumption that frost can cause ‘blue zones’ in tree ring chronologies during early xylogenesis (e.g., Kaennel and Schweingruber 1995). Such damages are not necessarily causing a collapse of cells. The images show cells that look normally differentiated, but still do not stain red in that zone. We assume that the spatial patterns within the damaged zones as well as the different degrees of damage among trees reflects the action of a ‘marginally critically low temperature’ under which small differences in developmental stage or in intrinsic freezing tolerance of such cells decide upon damage.

While early season freezing events can occur under all weather scenarios, an entire cool season or year is rare, and one that is cooler by 3 K then the mean is not in the meteorological records. According to Meteo-Swiss, 2009 was a normal-to-cool summer for the period 1990–2015, but with a Switzerland-wide annual mean of 5.8 °C the temperature was c. 1.3 K warmer than between 1945 and 1985, and nearly 2 K warmer than during the end of the ‘little ice age’ (1870–1910). Mean air temperature in June 2009 at the Stillberg site was 7.5 °C (the 1975–2010 mean is 7.6 °C), July was identical and August was 1 K cooler than the long-term average. Tree ring width for *P. uncinata* was 0.9 mm a^−1^ for 2009, which is the mean for the 2005–2016 period at Stillberg treeline (Möhl et al. 2018). Data for other sites in the Alps underpin that 2009 was an average year for tree growth at treeline across the past 4–5 decades (Wieser et al. 2018; Gruber et al. 2022). Below, we offer a potential physiological explanation for a lack of late wood lignification in response to an exceptionally cool late season that is not related to the lignification process per se, but to overarching (hormonal) controls of late season development.

## The blue-ring discussion in the literature

Blue zones can be embedded in tree rings among normally differentiated and lignified tissue. In the case shown by Büntgen et al. (2020; their Fig. 6), the blue zone was associated with a volcanic eruption in the year 536 that is likely to have caused an exceptionally cool growing season. Piermattei et al.’s (2015) analysis lines up with others, showing that such not freezing damage-related blue zones most commonly occur in the latest part of a tree ring, that is the last rows of cells produced in a season. In their example, cell walls in the late wood region looked well differentiated but still did not stain red in *Pinus nigra* (not a treeline species). These authors suggest a chronic cool period to cause this blue zone, but it could equally well have been a single event during that period that prevented these cells from proceeding toward proper lignification. By their meteorological minimum air temperatures of around 5 °C from a 600 m elevation station and the sites of sampling at around 2000 m (up to 2175 m), these trees must have experienced serious freezing during the late seasons that could produce a blue zone (cells inhibited from proceeding to lignification). Our data for the early season show that cells that were affected by a freezing event cannot resume the process of lignification when conditions improve.

The reported gradual decline in lignification in late wood, matches radio-densitometry results that show that cool late season conditions can lead to less dense and less lignified late wood in conifers (e.g., Schweingruber 1980; Gindl et al. 2000). We hypothesize the following mechanism for this late season blue zone formation. Unless a critical frost event comes into action, late wood maturation occurs under a conflict between the photoperiod-driven, late season transition to endo-dormancy (physiological hardening), and otherwise cool conditions that slow the rate of tissue maturation. Remarkably, rather warm summers (such as 1976) seem to increase the risk for such a mismatch (Greaves et al. 2022), since cell production continues later into the season under such warm conditions, as was shown by our + 3 K treatment by Lenz et al. (2013). The late season progression into dormancy and thus hardening, is a ‘life insurance’ mechanism that must come into action before life-threatening low temperatures occur. This gene-regulated developmental transition is likely to hold a higher hierarchy position of control than cell level mechanisms for reaching full lignification of the last cell rows of a season. These last cells can be prevented from completing lignification when cold events (slowing or sharply interrupting cell maturation) and photoperiod controls (accelerating the advance toward dormancy) come in conflict. However, it is important to note that the lignification process itself was not constrained by chronic 3 K cooling, and that sharp frosts can halt lignification in otherwise normally looking cells. As was mentioned in the introduction, a snow bed species can produce fully lignified xylem conduits even under constant 0 °C (Körner et al. 2019), conditions that would never occur in a non-dormant tree. Taken together, it is highly unlikely that lignification represents a biochemical bottleneck at low temperature that constrains wood formation more than do the other growth processes involved in cell wall formation under low temperature. The rare occurrence of poor or missing late wood lignification under exceptionally cool autumn weather, most likely reflects a developmental mismatch that can become enhanced by warm preceding weather.

## Conclusion

We suggest to make a distinction between effects of low-temperature extremes that can inhibit further cell differentiation, and create blue zones in tree rings (more likely early in the season) and more gradual looking declines in lignification at the end of the season, when photoperiod-driven transitions to endo-dormancy come in conflict with cold late season weather, either stopping (frost) or slowing (by cooling) the process of maturation. In our view, blue zones in late wood reflect such an interference of developmental controls (via hormones) at the whole tree level and ongoing metabolism in cell walls in some late season cell cohorts. So, it matters at which state of development cells are hit and thus, when exactly in a season such cold events occur. Whatever the reason, we agree on the usefulness of low-temperature-related blue zones in tree rings as additional markers in dendrochronology (Gindl et al. 2000; Büntgen et al 2022). Our in situ analysis of xylogenesis under an experimental climate, chronically 3 K cooler than at treeline, does not suggest that lignification is low-temperature limited in treeline trees. Except for the early season frost event we observed, tracheids of *Pinus uncinata* lignified normally also in the late wood of cold treated branch sections that experienced a temperature regime that corresponded to conditions at a 500 to 600 m higher elevation than that of the actual treeline position.

## Data Availability

There are no other data than the ones shown here and published in the companion paper by Lenz et al. (2013).
